# Long-term strategies to control COVID-19 in low and middle-income countries: an options overview of community-based, non-pharmacological interventions

**DOI:** 10.1007/s10654-020-00660-1

**Published:** 2020-07-13

**Authors:** Rajiv Chowdhury, Shammi Luhar, Nusrat Khan, Sohel Reza Choudhury, Imran Matin, Oscar H. Franco

**Affiliations:** 1 Department of Public Health and Primary Care, School of Clinical Medicine, University of Cambridge, Cambridge, United Kingdom; 2 Department of Epidemiology, National Heart Foundation Hospital and Research Institute, Dhaka, Bangladesh; 3 Brac Institute for Governance and Development, Brac University, Dhaka, Bangladesh; 4 Institute of Social and Preventive Medicine, University of Bern, Bern, Switzerland

**Keywords:** COVID-19, Low and middle-income countries, Non-pharmacological interventions, Exit plan, Zonal lockdown, Local lockdown, Rolling lockdown, Mitigation

## Abstract

In low and middle-income countries (LMICs), strict social distancing measures (e.g., nationwide lockdown) in response to the COVID-19 pandemic are unsustainable in the long-term due to knock-on socioeconomic and psychological effects. However, an optimal epidemiology-focused strategy for ‘safe-reopening’ (i.e., balancing between the economic and health consequences) remain unclear, particularly given the suboptimal disease surveillance and diagnostic infrastructure in these settings. As the lockdown is now being relaxed in many LMICs, in this paper, we have (1) conducted an epidemiology-based “options appraisal” of various available non-pharmacological intervention options that can be employed to safely lift the lockdowns (namely, sustained mitigation, zonal lockdown and rolling lockdown strategies), and (2) propose suitable application, pre-requisites, and inherent limitations for each measure. Among these, a sustained mitigation-only approach (adopted in many high-income countries) may not be feasible in most LMIC settings given the absence of nationwide population surveillance, generalised testing, contact tracing and critical care infrastructure needed to tackle the likely resurgence of infections. By contrast, zonal or local lockdowns may be suitable for some countries where systematic identification of new outbreak clusters in real-time would be feasible. This requires a generalised testing and surveillance structure, and a well-thought out (and executed) zone management plan. Finally, an intermittent, rolling lockdown strategy has recently been suggested by the World Health Organization as a potential strategy to get the epidemic under control in some LMI settings, where generalised mitigation and zonal containment is unfeasible. This strategy, however, needs to be carefully considered for economic costs and necessary supply chain reforms. In conclusion, while we propose three community-based, non-pharmacological options for LMICs, a suitable measure should be context-specific and based on: (1) epidemiological considerations, (2) social and economic costs, (3) existing health systems capabilities and (4) future-proof plans to implement and sustain the strategy.

## Introduction

The coronavirus disease-2019 (COVID-19) pandemic has claimed more than 500,000 lives worldwide [1] and has been responsible for significant economic disruptions globally [2]. Similar to the high-income nations, low and middle-income countries (LMICs) also responded to COVID-19 by implementing various population-level measures, including strict nationwide lockdowns and physical distancing [3]. Worldwide, with no effective treatments for COVID-19 and a vaccine at least a year away, these measures have been generally effective in preventing health systems from becoming overloaded, especially in the LMICs where: (1) the risk of disease transmission is high (populations are often large and dense, with a high degree of interaction and physical contact), (2) awareness of how to prevent disease is often poor (eg, clean water and hygiene practices), (3) public health systems are often under-resourced (eg, safety equipment and intensive care units/ICU), and (4) access to healthcare is limited and reliant on largely out-of-pocket payment.

These strict social distancing interventions, however, come with a price: they are unsustainable in the long term given their social, economic and psychological impacts. For example, a recently completed survey in Bangladesh showed that after its initial days of lockdown, a staggering 72% of urban and 54% of rural households had lost their main source of earnings [4]. Therefore, many LMICs are currently lifting the lockdowns, irrespective of the status of infection and the level of contagion. It remains, however, unclear what would be an optimal strategy for ‘safe re-opening’ (given the likelihood of disease resurgence), especially across low-income settings, where diagnostic capacities and surveillance infrastructure is poor [5].

**Fig. 1:**
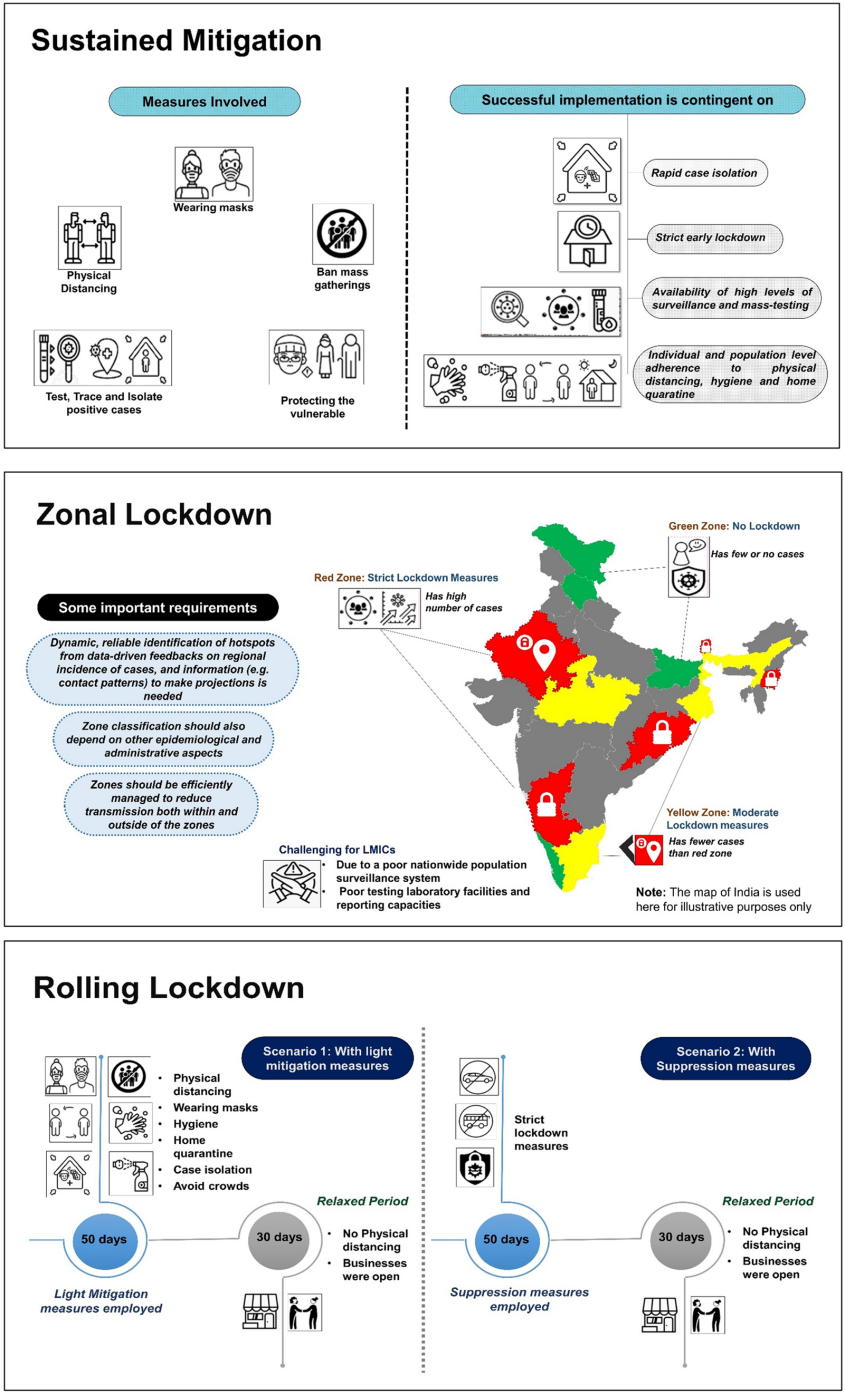
A visual summary of the three proposed community-based non-pharamacological option for developing countries

In this regard, we have considered three community-based non-pharmacological strategies for LMICs (which aim to strike a balance between health protection and preventing economic collapse) and propose appropriate application, ideal pre-requisites, and inherent limitations for each. They include: (1) sustained mitigation, (2) zonal lockdown, (3) rolling lockdown (dynamic measures). These strategies (as summarized in Fig. 1) should not be considered as mutually exclusive, and could be further adapted and combined depending on local disease epidemiology and socioeconomic circumstances.

## Sustained mitigation

Following the initial national lockdowns, staying on a ‘mitigation-only’ phase (a strategy adopted by developed countries such as France, Switzerland and Italy) has involved measures such as physical distancing, wearing masks, test-trace-isolation of suspected cases, shielding of the vulnerable and banning mass gatherings [6]. The successful implementation of this no-lockdown mitigation-only approach, however, is contingent on a number of key factors.

First, the implementation of the earlier strict lockdown has resulted in a significant reduction of contact rates, new infections, and case fatality in the country [7, 8]. In this regard, somewhat worryingly, many LMICs, where lockdown has recently been lifted, appear to have an upward trend of cases and deaths [9]. Second, there is availability of nationwide surveillance, mass testing operations and rapid case isolation to tackle any resurgence and to facilitate containment [10, 11]. Third, for contact-tracing, enough trained contact tracers (or scalable digital platforms) are available, with a relatively sparse target population (minimising the possibility of super-spreading events). In this regard, the effectiveness of contact-tracing might be importantly minimised in large, dense countries such as Bangladesh (~ 1300 people/sqkm), compared to sparsely populated countries like Spain (~ 90 people/sqkm) [12]. Contact-tracing is also less effective at the height of community spread when the rates are on the rise. Fourth, individual and population-level adherence to mitigation measures (eg, physical distancing, hygiene, home quarantine) will be ensured. For many LMICs, however, this remains a challenge given large-scale social stigma and suboptimal risk communication strategies [13]. Finally, healthcare services must be able to adequately cope with the resurgence in new cases, including availability of specialised care, hospitals and ICU beds. In many LMICs, there is however a chronic shortage of (1) critical care infrastructure (only 48,000 ventilators are available in India to serve its 1.3 billion people [14]), (2) personal protective equipment (PPE), (3) training of health workforce, and (4) good working conditions—all of which reduce system efficiency and enhance likelihood of transmission among healthcare workers.

Despite being far less restrictive than a full lockdown, a mitigation-only strategy is also not immune to financial hardship as it can lead to some socioeconomic disruption (e.g., reduced production due to workplace social distancing) – somewhat compromising its sustainability over a prolonged period. For example, Sweden adopted some of the most liberal mitigation measures in the world such as keeping restaurants, bars, and gyms open throughout the previous few months, whilst encouraging physical distancing rules. However, the country is still expected to suffer ~ 10% contraction in its economy in 2020 according to the Swedish Central Bank [15].

## Zonal lockdown

The idea of fencing between infected and healthy communities, termed *cordon sanitaire*, has been deployed during a variety of outbreaks for centuries. In line with this principle, as an exit strategy, many countries have transitioned to a system of “zonal (or local) lockdown” [16]. This system entails identification of specific “hotspots” where a sudden outbreak cluster, with a high number of cases, have been identified in real time. Such clustered social distancing works by dividing the population into “zones” according to the geospatial distribution of disease cluster contained within, so that interactions within a zone are significantly greater than interactions between zones [17]. Transmission hotspots, or “red zones” are subject to strict lockdown measures than “green zones”, where very few or no new cases have been identified for several days. Such strategies were adopted in France [17], with green zones defined by areas where the virus transmission is relatively low and there is not as burdensome pressure placed on the healthcare system.

The “zonal lockdown” approach has several important requirements. First, this categorisation of hotspots is typically a dynamic process, which requires an ability to reliably identify, in real time, areas that meet or fall short of the pre-specified lockdown criteria. This requires continuous data-driven feedbacks on: (1) regional daily confirmed cases (either by date of reporting or onset of symptoms), and (2) other time-series information needed to calculate the changes in region-specific effective reproduction number (*R,* the average number of secondary infections per infected individual), including daily numbers of hospitalized cases, daily numbers of deaths in different age groups, and transmission dynamics (eg, average time from infection to death) [17]. While such strategy has been successfully established in developed settings (such as France, where testing is widespread with 0.52 daily tests being done per 1000 population), this remains challenging in many LMICs due to (1) absence of large-scale population surveillance system based on randomly-selected individuals (e.g., in Bangladesh, the testing approach has focused on purposive, self-referred samples, with significant selection bias), and (2) poor testing laboratory facilities and reporting capacities (e.g., in Pakistan, only 0.09 daily tests are being conducted per 1000 individuals) [18]. In this regard, India has adapted a scalable mass "Pool testing" approach [19]. This cost-effective strategy involves collecting multiple samples in a tube and testing them with a single RT-PCR assay run. If the test is negative, all the people tested are negative. If it is positive, every person has to be tested individually for the virus. This approach reduces the time needed to test large swathes of the population [20].

Second, the classification of the zones should also be multifactorial. This should not only take into consideration the incidence rate, but also the other epidemiological (e.g., doubling rate of new cases; number of deaths) and administrative aspects (e.g., available hospital and ICU beds; testing and surveillance structure; residential versus industrial zone). Third, managing the zones efficiently to reduce transmission both within and outside of the zones is a major undertaking. Recent reports from India shows that infection size in many containment areas is 100-fold to 200-fold higher than the cases reported at those sites—indicating that containment efforts within zones may not have fully paid off [21]. Therefore, detailed *apriori* standard operating procedures should be devised to include aspects on (1) within-zone public health measures (eg, risk communication, house-to-house surveillance, test booths, contact-tracing, case referral systems, ambulance and medical facilities), (2) within-zone measures of emergency services (eg, food supply, law enforcement, isolation centres, and burial facilities), and (3) outside-zone measures such as creation of “buffer” zones (e.g., in India [19]) that surround the main containment zone to minimise out-of-zone transmissions. Such detailed protocols are crucial for efficiency. In Iran, for example, suboptimal zone management has increased risk of a second wave [22]. Finally, similar to sustained mitigation strategy, the zonal lockdown will be most effective when the overall rate of infection is in decline, accompanied by exhaustive vigilance.

While zonal lockdown, if implemented properly, can help contain the spread of the virus, efficacy of this approach can be reduced by other concurrent transmission networks, such as those linked to economic and social interdependency between zones [17]. Additionally, the impacts on the economy, particularly inside the zones, can be considerably more severe than under mitigation where the economy essentially opens with restrictions, exacerbating economic hardship in countries with already weak economic performance and social security nets. Therefore, these aspects merit careful consideration during the planning phase of this strategy.

## Rolling lockdown

Intermittent or “rolling” lockdown measures take place when strict social distancing measures are applied and lifted periodically. This strategy has been described as a potentially effective measure to minimise uncertainty in both effective *R* values, and in the severity of the virus (i.e. the proportion of cases requiring ICU admission) [23]. This approach may be particularly suitable for the LMICs with large and dense populations, high patterns of contact, poor economic/health systems resilience, and weak testing/contact tracing capacities. Furthermore, this approach addresses both key elements of society that needs safe-keeping: life and livelihood, and aims to provide a balance between avoiding public health systems being overloaded and grinding economies completely to a halt [24]. A recent paper mathematically modelled the effects of either a strict 50-day suppression or a 50-day mitigation, followed by 30 days of relaxation (during which businesses are allowed to reopen, with basic hygiene measure kept in place), in 16 economically diverse countries. In these models, a strict 50-day lockdown, that reduces the effective *R* value to 0.5, prevented ICU beds overload and led to considerably fewer deaths (130,000 during 18 months in the 16 countries they modelled) compared to a more relaxed 50-day mitigation/30-day relaxation cycle (~ 3.5 million predicted deaths globally) and under no-intervention (counter-factual) scenario (8 million predicted deaths) [25]. To further contextualize the value of such concept, a subsequent paper estimated that (1) a single, one-off lockdown will be insufficient to bring the pandemic under control, and (2) secondary peaks would be larger than the first, without continued restrictions [26].

However, as with the other strategies, rolling lockdown approach is also contingent on several factors. First, before implementing a rolling lockdown, every developing country should carefully consider the economic and social costs to implement these measures. Second, impacts on incidence and case-fatality will rely on local levels of adequate adherence to social distancing measures. Third, this approach would also bring a new set of logistical challenges. Therefore, countries will need to formulate bespoke plans for reorganising business supply chains, so that they align with the economy opening and closing. While such readjustments to complement a schedule of lockdown is not ideal, unprecedented challenges often require unusual and adaptive solutions, especially if other alternative exit strategies are not feasible. Finally, by establishing a detailed surveillance system while the lockdown takes place, countries should adapt the duration of the lockdown and relaxation periods according to the local growth rate and pattern of the epidemic. A recent example of this has been in Pakistan, where the World Health Organization has recommended a 14-day-on/14-day-off rolling lockdown to control the epidemic [27]. Similarly, rolling lockdowns do not have to be generalised, these can also be adapted as regional or zonal rolling lockdowns within a country, i.e., to apply specifically in areas with high and sustained new-onset COVID-19 cases per population. For example, zonal rolling lockdowns have been proposed in the Gauteng province of South Africa—one of the worst affected regions in the country—to control the rapid increase in infection rates [28]. 

## Conclusion

While many LMI countries are currently lifting the lockdowns due to economic reasons, it is crucial for the policy makers to recognise that preserving health is equally important for reviving the economy. This is of important relevance to the LMICs where large proportions of working-age population are vulnerable to adverse COVID-19 outcomes, owing to high prevalence of comorbid conditions (such as diabetes, obesity and hypertension) [29]. Furthermore, if a country has constant high incidence of a deadly disease, it may become rather challenging for the local economy to thrive in such environment [30]. Therefore, equal priorities must be put on protecting lives as well as livelihood when adapting an exit plan. In this regard, we have proposed several non-pharmacological strategies that may enable the LMICs to safely open the economy, while allowing for preservation of health. However, it is crucial that the selection of a suitable, “context-specific” strategy is based on some key considerations: (1) local epidemic growth rate, (2) existing health infrastructure (to survey, test, and treat, at scale), (3) social and economic costs, and (4) carefully-devised plans to implement and sustain the measures.
